# Thymus Gland: A Double Edge Sword for Coronaviruses

**DOI:** 10.3390/vaccines9101119

**Published:** 2021-10-02

**Authors:** Ebtesam A. Al-Suhaimi, Meneerah A. Aljafary, Fadwa M. Alkhulaifi, Hanan A. Aldossary, Thamer Alshammari, Ayman AL-Qaaneh, Razan Aldahhan, Zahra Alkhalifah, Zagit Z. Gaymalov, Adeeb Shehzad, Abdelgadir M. Homeida

**Affiliations:** 1 Biology Department, College of Science, Imam Abdulrahman Bin Faisal University, P.O. Box 1982, Dammam 31441, Saudi Arabia; maljafary@iau.edu.sa (M.A.A.); falkhulaifi@iau.edu.sa (F.M.A.); amhomeida@iau.edu.sa (A.M.H.); 2 Epidemic Diseases Research Department, Institute for Research and Medical Consultations, Imam Abdulrahman Bin Faisal University, P.O. Box 1982, Dammam 31441, Saudi Arabia; 2200500232@iau.edu.sa or; 3 Genetic Research Department, Institute for Research and Medical Consultations, Imam Abdulrahman Bin Faisal University, P.O. Box 1982, Dammam 31441, Saudi Arabia; tmjalshammari@iau.edu.sa (T.A.); Ayman.qaaneh@JHAH.com (A.A.-Q.); 2200500002@iau.edu.sa (Z.A.); 4 Clinical Pharmacy Services Division, Pharmacy Services Department, Johns Hopkins Aramco Healthcare (JHAH), Dhahran 31311, Saudi Arabia; 5 Stem Cell Research Department, Institute for Research and Medical Consultations, Imam Abdulrahman Bin Faisal University, P.O. Box 1982, Dammam 31441, Saudi Arabia; raaldahhan@gmail.com; 6 Earlystage OÜ, Lasnamäe Linnaosa, Sepapaja tn 6, Harju Maakond, 15551 Tallinn, Estonia; z@earlystage.co; 7 Clinical Pharmacy Research Department, Institute for Research and Medical Consultations (IRMC), Imam Abdulrahman Bin Faisal University, P.O. Box 1982, Dammam 31441, Saudi Arabia; asmsiar@iau.edu.sa

**Keywords:** hedging, transaction costs, dynamic programming, risk management, post-decision state variable

## Abstract

The thymus is the main lymphoid organ that regulates the immune and endocrine systems by controlling thymic cell proliferation and differentiation. The gland is a primary lymphoid organ responsible for generating mature T cells into CD4+ or CD8+ single-positive (SP) T cells, contributing to cellular immunity. Regarding humoral immunity, the thymic plasma cells almost exclusively secrete IgG1 and IgG3, the two main complement-fixing effector IgG subclasses. Deformity in the thymus can lead to inflammatory diseases. Hassall’s corpuscles’ epithelial lining produces thymic stromal lymphopoietin, which induces differentiation of CDs thymocytes into regulatory T cells within the thymus medulla. Thymic B lymphocytes produce immunoglobulins and immunoregulating hormones, including thymosin. Modulation in T cell and naive T cells decrement due to thymus deformity induce alteration in the secretion of various inflammatory factors, resulting in multiple diseases. Influenza virus activates thymic CD4+ CD8+ thymocytes and a large amount of IFNγ. IFNs limit virus spread, enhance macrophages’ phagocytosis, and promote the natural killer cell restriction activity against infected cells. Th2 lymphocytes-produced cytokine IL-4 can bind to antiviral INFγ, decreasing the cell susceptibility and downregulating viral receptors. COVID-19 epitopes (S, M, and N proteins) with ≥90% identity to the SARS-CoV sequence have been predicted. These epitopes trigger immunity for antibodies production. Boosting the immune system by improving thymus function can be a therapeutic strategy for preventing virus-related diseases. This review aims to summarize the endocrine-immunoregulatory functions of the thymus and the underlying mechanisms in the prevention of COVID-19.

## 1. Introduction

The coronavirus family was first identified in the late [1]. In the decades since, the world has experienced many lethal episodes of the coronavirus family. Coronavirus diseases were noted with mild or severe infections in the respiratory tract [2]. In 2002, severe acute respiratory syndrome coronavirus (SARS-CoV) emerged and infected many populations worldwide [3]. In 2012, the Middle East respiratory syndrome (MERS-CoV) outbreak infected Middle Eastern countries, with symptoms of chronic respiratory syndrome [4]. In 2019, SARS-CoV-2 was identified in Wuhan, China, which affected a total of 30,524,214 people with a mortality of 952,240 at the time of drafting this paper. SARS-CoV, MERS-CoV, and SARS-CoV-2 involve serious respiratory tract infections followed by fever, cough, dyspnea, and fatigue [5].

The thymus gland is the chief lymphoid organ that regulates the functions of the immune and endocrine systems by controlling the levels of hormones and cytokines. The thymus gland protects against various internal and external stresses through immunoregulatory properties, nerve systems, and endocrine pathways. The thymus gland controls cell proliferation, apoptosis, hormones, and neuropeptides, as well as regulating intrathymic T cell differentiation and production of a repertoire of the T cell. The thymus is located in front of the heart behind the sternum. It has two identical thymic lobes on each side, made up of the cortex and the central medulla, surrounded by an outer capsule [6]. The thymus gland is most actively functioning in fetal and neonatal life and starts shrinking in tissue mass and is replaced with fat during thymic involution [7]. In 1961, Jacques Miller discovered the immunoregulatory role of the thymus in newborn mice by studying involvement in a lymphocyte population [8].

The reason for children being less exposed to SARS-CoV-2 could be attributed to the significant capacity of children for maintaining the availability of rare T cell clonotypes that originate in the thymus and the variety of the T cell populations, which supposes a causative connection in the rising tendency of infection with age [9,10]. This clonotype is rare if it is not utilized, which leads to less proliferation and postpones presenting viral antigens to SARS-CoV-2-specific T cells, permitting more effects of virus damage and breakout. This late activation of the adaptive power in the immune system appears as lymphopenia in lack of effective virus-specific clonal growth specific to epitopes of the virus presented by the lymphatic nodes. It was suggested by Rousseau et al. (2020) [9] that stimulating the same approach will help in reducing the severity of this virus. Thymic hormones such as thymosin-α-1-Fc (TA-1) have been shown to increase the naive CD8 and CD4 cells that have recombined in the blood and stimulate thymopoiesis, then TA-1 adjusts a hyperinflammatory response via dendritic cells (DCs) for immunosuppressing and activating natural killer (NK) cell function. The absence of type 1 interferon (IFN) in alveolar cells and the presence of a lymphopenia response in SARS-CoV-2 diseases propose that incorporation of αβ-IFN and TA-1 may present the synergistic action to attract the adaptive immunity that helps significantly in a much-needed response.

The immune system is generally classified into innate immunity which provides the first line of defense against different stimuli, such as antigens and chemical, biochemical, physiological, and physical stresses. Cellular innate immunity mediates their actions through macrophages, granulocytes, NK cells, and DCs, either by engulfing the antigens in a process called phagocytosis or by acting as antigen-presenting cells to expose the antigens to the cells of the acquired immunity, which is a more specific type of immunity. B lymphocytes and T lymphocytes are interrelated cellular counterparts of the acquired immune system, expressed as surface receptors that recognize specific antigens and have the potential for long-term immunological memory. Lymphocytes are generated in the bone marrow, where only B lymphocytes mature and are exported to the periphery. However, T lymphocytes, as hematopoietic precursors, migrate to the thymus to grow, develop, and differentiate [11].

The thymus offers specialized conditions for developing various functional and self-tolerant T cells. Once in the thymus, precursor cells enter the subcapsular cortical region, undergoing several developmental stages to become thymocytes. In the differentiation process, thymocytes move from the cortex area to the medulla for negative and positive thymic selection [12]. Thymocytes interact with the thymic epithelial cells and trigger their differentiation into mature clusters of differentiation 4 (CD4+) and CD8+. Cytotoxic T lymphocytes (CTLs) undergo a process known as the positive selection, where thymocytes recognize and bind to self-peptides of the major histocompatibility complex (MHC). This process determines whether the T lymphocyte is CD4+ (helper) or CD8+ (cytotoxic/killer), depending on bonding to the type of MHC (class I or class II). When self-reactive thymocytes fail to recognize self-antigens and strongly bind to the self-peptide MHC, they undergo negative selection and are eliminated by the process of apoptosis [11]. The naive T lymphocytes disembark the thymus into the secondary lymphoid organs such as lymph nodes, where they are activated by foreign peptides of MHC that are found on the surfaces of the antigen-presenting cells (APCs). This type of activation results in the proliferation and differentiation of effector T lymphocytes into four types that can produce cytokines and respond to different pathogens. The thymus gland also secretes the thymosin hormone, which has a functional role in T lymphocyte differentiation and maturation to mediate immunological response [13]. T lymphocytes are the most fundamental components of cellular immunity. Besides humoral immunity, they also have featured roles as a complementary component to innate immunity, which cannot efficiently defend against all pathogens.

**Figure 1:**
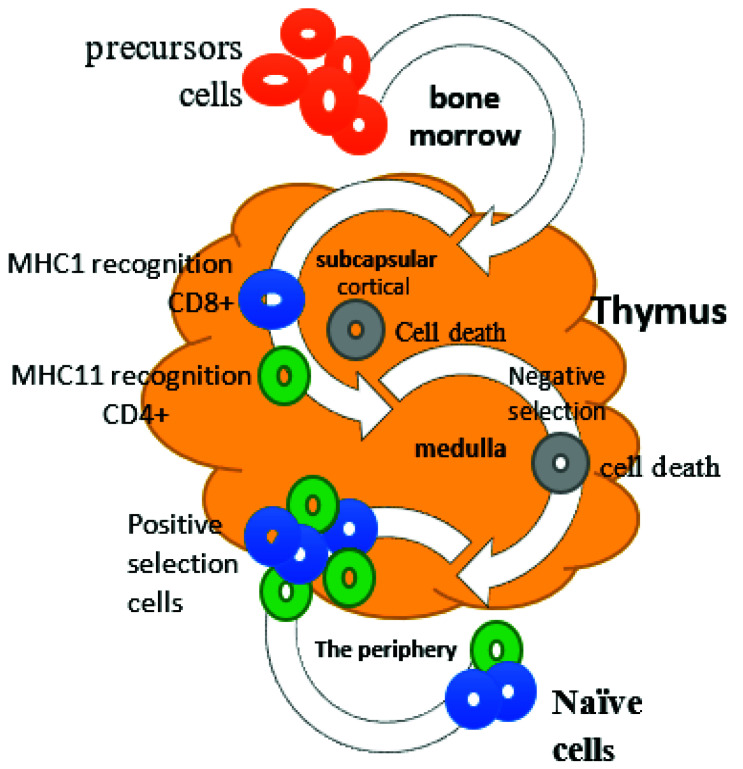
Overall role of the thymus gland in the development of T cells.

Some immunodeficiency viral diseases such as thymic lymphoid hyperplasia (thymitis), loss of Hassall’s corpuscles, and dysinvolution have been associated with the malfunctioning of thymus or viral infections of Hassall’s corpuscles [14]. Hassall’s corpuscles were also found to be severely damaged upon infection with herpes simplex virus pneumonia and ependymoma [15]. SARS-CoV induces immune-mediated lymphocyte damage, bone marrow or thymus suppression, or cell programming death [16]. The SARS-CoV-2 infection causes lymphopenia in peripheral circulation, which is counterbalanced by the thymus to enhance lymphocyte recirculation between peripheral blood and SARS-specific IgG immunoglobulins release, with no elevation in the levels of interleukin (IL) 8 and tumor necrosis factor (TNF-α) [16,17]. The aim of the current study is to support the notion that a defect in thymic tolerogenic function is implicated as an essential factor in the pathophysiology of autoimmunity and virus-related diseases, including COVID-19. This piece of work underpins the reported literature on the physiology of the thymus (Figure 1) and the biological role of different thymic hormones with regard to the modulation of inflammatory responses and involvement in the maturation and differentiation of immune cells, as well as advocating the clinical and biological application in the treatment of inflammatory disorders, including viral diseases.

## 2. Cellular Immunity and Role of T Lymphocytes

**Figure 2:**
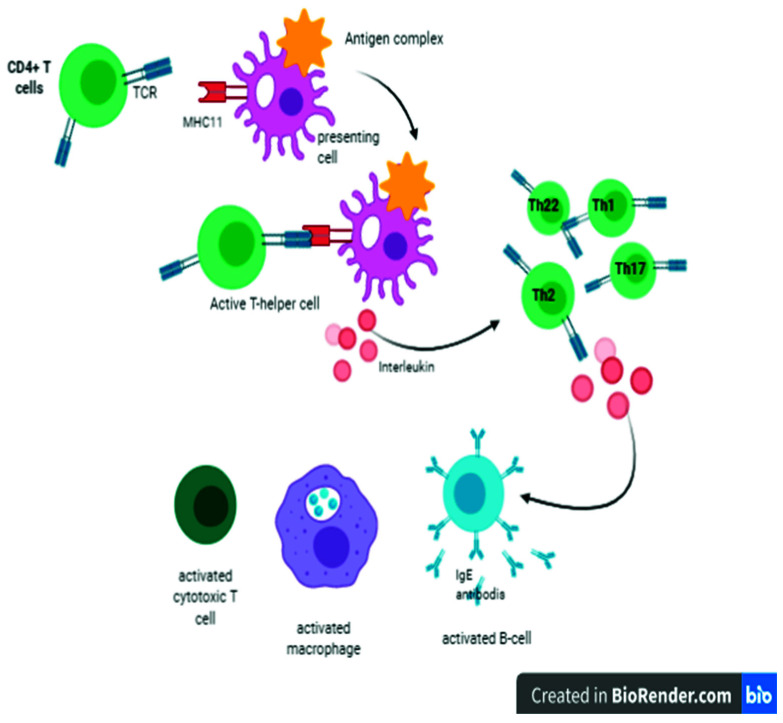
Antigen-presenting cells present an antigen complex with a major histocompatibility complex (MHC) to stimulate immature T cells to become either cytotoxic cells (CD8), when the T cell receptor binds to MHC class I, or Th cells (CD4+), when it binds to MHC class II. Once CD4 cells are activated, they will begin proliferation or clonal expansion and differentiate into Th17, Th9, Th1, Th2, and Th22 at the same time; they will secret interleukins that will stimulate a humoral immune response to produce antibodies (IgE), as well as cellular immune response and nonspecific defense by activated cytotoxic T cells and macrophages.

There is compelling evidence that the thymus is responsible for the development and differentiation of T lymphocytes. T lymphocytes mediate cellular immunity, providing a defense mechanism against intracellular microorganisms either through the direct killing of the cells that host microorganisms in their cytoplasm or the activation of other immune cells to destroy ingested microorganisms by the process of phagocytosis and productions of antibodies against specific antigens [18,19]. It has been reported that short chains of peptides that bind to MHC-I molecules are derived from degraded intracellular cytosolic proteins of microorganisms, including virus-mediated infections. On the other hand, peptides that bind to MHC-II molecules are derived from extracellular proteins of infectious agents [20]. The CD4+ T lymphocytes recognize antigens presented by MHC-II molecules, readily expressed on DCs, macrophages, and B lymphocytes [20]. Consequently, CD4+ T lymphocytes are activated and differentiated into one of several subsets of effector T helper (Th) lymphocytes, including, Th1, Th2, Th9, Th17, and Th22, which regulate the immune responses (Figure 2) by secreting cytokines [21]. Th2 lymphocytes also regulate the production of antibodies from B lymphocytes by secreting ILs such as IL-3, IL-4, and IL-5 and differentiation to antibody-secreting plasma cells [22]. Th17 has been reported to prevent mucosal respiratory viral infections in the lungs through recruiting macrophages and neutrophils, which ultimately clears pathogens, mediating inflammation, and maintaining tissue integrity [23]. Th17 lymphocytes have suppressed the detrimental tissue inflammations in viral infections. The exact underlying defensive mechanism of Th17 lymphocytes remains to be elucidated because lymphocytes-associated viral lung pathologies have been reported previously [24].

It is well known that inflammation is a central player in the pathogenesis of SARS-CoV pneumonia and edema [25]. Moreover, Th1 lymphocytes are responsible for the proliferation and differentiation of cytotoxic CD8+ T lymphocytes and other cells in response to intracellular pathogens, as well as latent viral infections and tumors, which are all weak innate immune response inducers. Various cytokines such as IL-2, IL-12, IL-15, and IL-21 are involved in CD8+ T lymphocyte differentiation and the generation of effector and memory lymphocytes. CD4+ Th lymphocytes promote CD8+ T lymphocyte activation, either directly by cytokine production or indirectly by enhancing the ability of APCs to stimulate the activation process [19]. CTLs recognize the cells presenting MHC-I molecules, which serve as legends to the T-cell receptors (TCRs) on their surfaces, targeting them for destruction. In this process, only antigen-expressing cells are affected and destroyed. CTLs activate macrophages through interferon gamma (IFNγ) production, which can phagocytose microorganisms [26]. The activity of Th lymphocytes and CTLs is regulated by the regulatory T lymphocytes (Treg), known as suppressor T lymphocytes, and a subtype of CD4+ T lymphocyte. Treg lymphocytes account for 5–10% of CD4+ T lymphocytes in the periphery and play an essential role in inhibiting autoimmune and chronic inflammatory diseases. Treg cells also eliminate self-reactive T lymphocytes that have escaped central tolerance (negative thymic selection) by the mechanism of peripheral tolerance [27]. Once a pathogen or a disease-causing agent is identified, naive T lymphocytes proliferate and differentiate into effector T lymphocytes, ultimately targeting and eliminating the foreign invaders. Effector T lymphocytes also serve as memory T lymphocytes, such as stem cell memory T lymphocytes (Tscm), central memory T lymphocytes (Tcm), and effector memory T lymphocytes (Tem), as well as terminal effector T lymphocytes and tissue-resident memory T lymphocytes, which can respond faster and more efficiently in the future against the same infection [28]. Compared with naive T lymphocytes, Tscm cells reveal higher expression levels of C-X-C motif chemokine receptor 3 (CXCR3), apoptosis antigen-1 (APO-1), IL-2 receptor (IL-2Rβ), and leukocyte function-associated antigen-1 (LFA-1). Tem and Tcm cells are differentiated by the function and expression of the C-C chemokine receptor type-7 (CCR7) protein. Tcm cells are occupied in the lymphoid system and have no direct role, while Tem cells are found in the non-organ lymphatic tissue and have a rapid and more significant function than Tcm cells [29].

## 3. Function of Hassall’s Corpuscles in Viral Infection

There is compelling evidence that thymic stromal lymphopoietin (TSLP) is a cytokine, which stimulates B lymphocytes development derived from thymic stromal cell line Z210R.1. It has been reported that Hassall’s corpuscles’ epithelial cells produced TSLP, responsible for the activation of thymic DCs to induce and generate CD4+ CD25+ regulatory T cells within the thymus. In addition, Hassall’s corpuscles are accountable for thymocyte development and removal of apoptotic thymocytes inside the thymus [30]. The primary extravillous trophoblast (EVT) expressed the cytokine TSLP and TSLP receptors. Studies have shown that TNF-α and IL-4 or pregnancy-associated hormones lead to a substantial rise in TSLP-mediated primary human EVT propagation and invasion in vitro. TSLP has a crucial role in human EVT invasion and regulation of the placenta in the first trimester of pregnancy [31].

Transforming growth factor alpha (TGF-α) is associated with medullary human thymic epithelial cells (TECs) and thymic Hassall’s corpuscles, whereas epidermal growth factor (EGF) receptor was concentrated only in TEC cells through the thymus tissue [32]. Both TGF-α and EGF are crucial regulatory precursors for synthesizing TEC-derived cytokines in the thymus and act as essential modulators for developing T lymphocyte proliferation in humans [33]. TGFβRII was found to mediate TEC signaling and reduce their improvement in Hassall’s corpuscles in mice [34].

**Figure 3:**
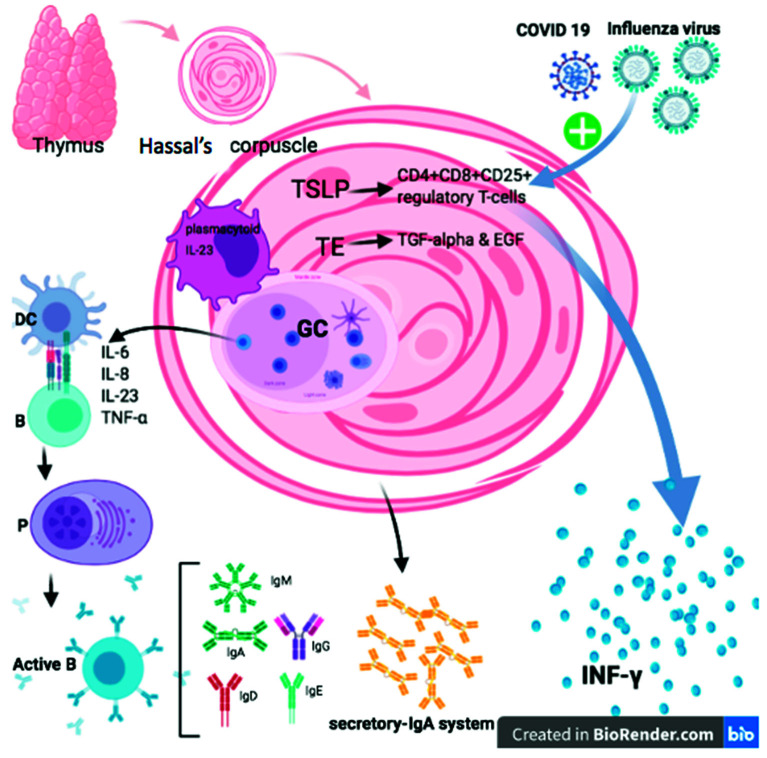
Functions of thymic Hassall’s corpuscles in normal state and after infection with influenza virus. Thymic stromal lymphopoietin (TSLP), a cytokine that is the primary hormone produced by the epithelial cells of Hassall’s corpuscles, was found in the thymus and was responsible for the activation of thymic dendritic cells. Hassall’s corpuscles secrete TSLP-inducing dendritic cells to induce and generate CD4+ CD8+ CD25+ regulatory T cells within the thymus. Thymic plasmacytoid dendritic cells express IL-23 receptors. Transforming growth factor alpha (TGF-α) was identified in medullary human thymic epithelial (TE) cells and thymic Hassall’s corpuscles while epidermal growth factor receptor (EGF-R) was concentrated to TE cells through the thymus tissue. Hence, TGF-α and EGF are crucial regulatory precursors for the synthesis of TE cell-derived cytokines in the thymus. The human thymus shows the existence of antibodies IgG, IgA, IgM, IgD, and IgE, which are secretory constituents in Hassall’s corpuscles. There is a strong connection between the amounts of IgA and secretory components in the cells of Hassall’s corpuscles, and the thymus may have to be considered as an active portion of the secretory-IgA system of Hassall’s corpuscles. The influenza virus activates thymic CD4+ CD8+ thymocytes, leading to the secretion of a large amount of interferon IFNγ.

Hassall’s corpuscles are composed of terminally differentiated medullary TECs with properties of cellular senescence and release inflammatory cytokines and chemokines, such as CXCL5, that employ and activate neutrophils to release IL-23 in the thymic medulla. Thymic plasmacytoid DCs express IL-23 receptors essentially produce IFNγ, which functions in cell maturation [35]. The human thymus expresses antibodies IgG, IgA, IgM, IgD, and IgE, and light chains, in the cells of Hassall’s corpuscles. In the thymic medulla, the production of IgG, IgA, and IgM by plasma cells is controlled. (Figure 3) [36].

IL-1α/β enhances IgM and recruitment of CD4+ T cells at the site of infection but does not contribute to killing the virus-infected cell [37]. Additionally, CD4+ but not CD8+ Treg cells were suppressed by IL-6, allowing pathogen clearance and host survival in virus-induced infection [38]. In H1N1 influenza, IL-6 levels elevated in severe cases of virally infected patients [39] and activated CD4+ CD8+ thymocytes and a large amount of IFNγ [40]. While IL-32 is a part of a negative feedback loop, inhibiting sIL-6R and upregulating IL-6 is essential for the survival of an influenza A virus infection [35,41]. Few immunodeficiency viral diseases have been linked with the thymus in childhood, including thymic lymphoid hyperplasia (thymitis), loss of Hassall’s corpuscles, and dysinvolution [14]. Hassall’s corpuscles were found to be altered and damaged in a 4-year-old boy infected with herpes simplex virus pneumonia and ependymoma [15]. Modulation in the size of the thymus has been associated with hyperactivation of dystrophic calcification of Hassall bodies, reflecting the decrease in the number of CD4+ cells in drug-addicted patients [42]. Lymphocytopenia is a noticeable portion of SARS-CoV contagion. It may be immune-mediated lymphocyte damage, bone marrow or thymus suppression, or cell programming death [16]. After a viral infection, lymphopenia is noticed in peripheral circulation [17].

## 4. Role of Thymic Hormones in Viral Infection

**Figure 4:**
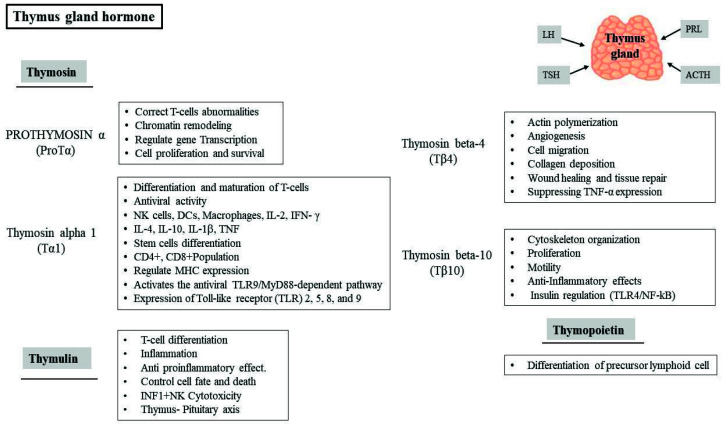
Types and roles of thymus gland hormones. The thymus produces immunoregulating hormones such as thymosin, and its family includes prothymosin alpha, thymosin alpha 1, thymosin beta-4 and thymosin beta-10, thymuline, and thymopoietin.

The thymus is a lymphoid organ involved in T lymphocyte maturation and differentiation. It is known that TECs can secrete IL-6 and granulocyte-macrophage colony-stimulating factor (GM-CSF) and thymic hormones in circulation, which promote thymocyte differentiation and proliferation and have anti-inflammatory effects. Thymic hormones (Figure 4) such as thymopoietin, thymosin alpha 1 (Tα1), and thymuline have a potential role in the differentiation and functions of lymphocytes, thus they may have the potential for T lymphocyte-related diseases. In addition, the thymus mediates neuroendocrine interactions directly affected by pituitary hormones, consequently affecting the neuroendocrine function of the thymus [43]. The active biological thymic peptides have been extracted and purified in a process called Thymosin Fraction V, along with several main peptides such as prothymosin α (ProTα), Tα1, thymosin beta-4 (Tβ4), thymosin beta-10 (Tβ10), and thymuline, for the maturation and differentiation of immature thymocytes [44,45,46]. These peptides are biologically essential and known to activate the immune system through several mechanisms and signaling pathways, including stimulation of T cell differentiation and maturation, activation of NK cells, DCs, and induction of proinflammatory cytokine release [47]. The previous description indicates that thymic hormones can mediate anti-inflammatory effects, and future clinical trials are needed to translate them against inflammatory disorders and viral diseases [48]. In an experimental model of allergic asthma, a dose of DNA nanoparticles, including thymuline plasmids, could protect the lungs from some injurious inflammation and muscular hypertrophy, which recovered respiratory mechanical functions [49]. Mice were treated through intratracheal administration with a dose of thymuline-expressing plasmids administrated with nanoparticles to enable the thymuline to infiltrate the mucus barrier of the respiratory system [50].

**Figure 5:**
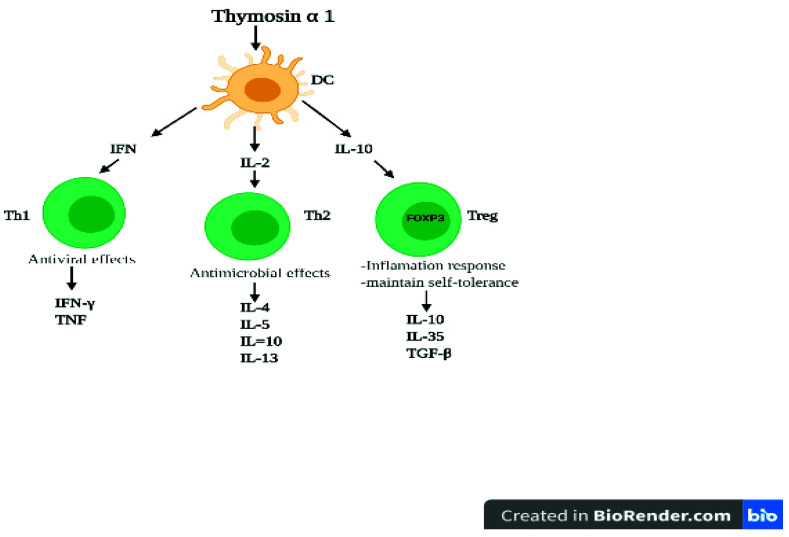
T alpha 1 cells and functional outcomes by targeting dendritic cells. Tα1 cells can modulate dendritic cell (DC) function. DCs express variable receptors for communications to induce T helper type 1 (Th1), T helper type2 (Th2), and regulatory T cells (Treg) priming antigen-specific T cell activation. Tα1 cells convert resting DCs into cells capable of promoting the polarization and differentiation of naive T cells.

The most important member of the thymosin family is Tα1 (Figure 5) and its precursor ProTα [48].

Tα1 is highly expressed in the thymus and peripheral tissues and produced through cleavage of ProTα in the thymus, pituitary, and brain [51]. Thymus hormones are targeted to control viral infectious diseases and inflammatory and autoimmune diseases [46,52]. Tα1, Tβ4, and Tβ10 displayed positive immunomodulatory effects by inducing Th lymphocytes (CD4+) and activating cytotoxic T lymphocytes (CD8+), maintaining immune homeostasis in viral infection [53].

**Figure 6:**
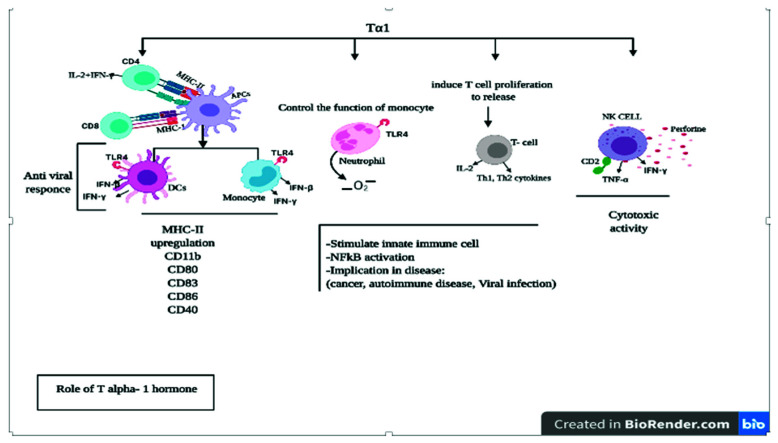
Effect of Tα1 on cells and pathways of the immune system. Tα1 can trigger multiple downstream pathways with distinct types of immune cells, pro/anti-inflammatory cytokine, and induce an immune response by Toll-like receptor (TLR) expression and cytokine production, leading to the initiation of a subsequent phase of immunity.

Tα1 boosts immunity through the differentiation and maturation of T cells, and the activation of NK cells, DCs, and release of proinflammatory cytokines [47] (Figure 6). The pharmacological and immunomodulatory effects of Tα1 have been investigated in various animal and human studies, including the treatment of chronic hepatitis B and C, cytomegalovirus infection, sepsis, a chronic obstructive pulmonary disorder, HIV/AIDS, and SARS-CoV [46,54,55]. The principal effector cells in the innate immune system are DCs, NK cells, and NK T cells, with monocytes, macrophages, and Tα1 as a biological response modifier, which regulates the differentiation and maturation in viral infections [52]. Tα1 modulates T lymphocytes and was used as a prophylactic agent against the SARS virus throughout the 2003 pandemic. Therefore, it can control immunity, inflammation, and the development of the disease [56]. Tα1 is widely used as a therapeutic agent in viral, fungal, and bacterial infectious diseases, either as a monotherapy or a vaccine enhancer, as an adjuvant with IFNα [48,52]. Under normal conditions, transformed cells can upregulate and increase the expression of MHC-I, MHC-II, and macroglobulin (B2) [47], generally required for recognition by the immune system of the virally infected cells, and thus can directly blunt the growth of viruses [57]. Additionally, when mononuclear cells are treated with ProTα, there is an increased expression of IFNα-inducible protein, which possesses significant antiviral activity [58]. Mujtaba et al. recommended using re-designing antivirals and important potential inhibitors against COVID 19 [59]. Tα1 can modulate both T lymphocyte maturation and NK cell-mediated cytotoxicity, as well as stimulation of lymphokine and cytokines production by peripheral blood lymphocytes, which include macrophage migration inhibitory factor, CSF, GM-CSF, B lymphocyte growth factor, IFNα, IFNγ cascade, and IL-2 by activated lymphocytes. Tα1 also regulates transcriptional factors involved in the immune response and influencing protein/antigen expression [60]. Tα1 regulates Th1-type cytokines that may affect thymocytes by stimulating their differentiation, converting them to active T lymphocytes, and enhancing NK cell activity [61]. When immature DCs (iDCs) in the periphery blood are activated by pathogenic determinants known as pathogen-associated molecular patterns or by immune cytokines (TNF-α and IL-1β), iDCs become activated and transformed into fully mature DCs (mDCs) by upregulating co-stimulatory molecules (CD-40, CD-86, CD-80, and CD-83) followed by overexpression of IL-12 [62]. There is compelling evidence that Tα1 modulates mitogen-activated protein kinases (MAPKs) and signals the transduction pathway to activate bone marrow-derived macrophages [63] and mDCs through interaction with Toll-like receptors (TLR) by the MyD88-dependent pathway, in particular TLR2, TLR5, TLR8, and TLR9. It is known that mDCs migrate to lymph nodes and macrophages, where they interact with numbers of naive T lymphocytes. DCs increase antigen presentation and synapses with CD4+ helper T lymphocytes and Th cytokines (e.g., IL-2, IFNγ) [64]. Therefore, activating some intracellular signaling pathways such as NF-kB and p38 MAPK are required for the therapeutic efficacy of thymus hormones [47,65,66]. IFN-I plays a vital role in mediating T cell response at the site of viral infection. Moreover, IFN-I can stimulate genes that promote autophagy and boost immunity against viral infections [67]. Studies have shown that in MERS-CoV, macrophages are primary effectors of the innate immune system and DCs are present abundantly in infected lungs. They control inflammatory cytokine producers and APCs by direct interaction with the antigen and surface and the intracellular receptors [68]. This causes the production of interferon gamma-induced protein 10 (CXCL10), mRNAs, and IFN (IFNλ1, IFNα/β expression), which can enhance the production of CD8+ T lymphocytes and cytolytic functions by producing perforin and granzyme [69]. This inhibits viral infection through direct cytotoxicity or by proinflammatory cytokine productions [53,67].

## 5. Cytokines Activity against Coronaviruses

The key function of innate immunity has a known role in leading both local and systemic inflammation and the release of the cytokine-causing storm in the acute respiratory infection of COVID 19. This immune response dysfunction is caused by interferon (IFN) components and activation of the complement system that progress inflammations and lung tissue injury. IFNs regulate viral contagion by upregulating the expression of IFN-stimulated genes that straiten some phases of virus replication [70]. Type I interferon (IFN-I) has a significant role in the host’s innate immunity; as an innate immunity barrier, its function is to exclude the virus once virus infection happens in its early phase to prevent its replication. However, SARS-CoV-2 has developed multiple protective plans to escape from innate immunity response by using nonstructural and structural proteins and accessory proteins as IFN-I antagonists to allow virus replication, contagion, transmission, and finally pathogenesis [71]. Therefore, targeting INFs and their antagonists may help in COVID management. Nakhlband et al. reported that early treatment with INF-α can be considered as a promising therapeutical plan for COVID-19. Both human interferon alpha 2 (IFN α 2) and thymosin alpha 1 (T α 1) are curative proteins applied against viral infections and several kinds of cancer. Both IFN α 2 and T α 1 display a synergic action in their efficiency as a fusion protein (in combination) [72]. In an in vitro study, IFN α 2-T α 1 is more active than monocular IFN α 2 as an antiviral and anticancer treatment [73].

Along with the role of thymic hormones on T lymphocyte maturation, the expression of different cytokines also determines the activity and functionality of immune cells. The antipathogenic effect of IFNs is facilitated through contact with TLRs, which inhibits the viral replication inside the host. When a virus invades the host cell, pattern recognition receptors identify the viral nucleic acid and activate transcription factors such as interferon regulatory factors IRF3 and IRF7, translocating to the nucleus for the production of IFN1. Along with this, it activates downstream targets Janus kinase/signal transducers and activators of the transcription signal pathway, which enhances the expression of IFN-downstream targets. IFNs can limit the virus spread by enhancing the phagocytic activity of macrophages against the virally infected cells and promoting the NK cell control activity against infected target cells. Therefore, inadequate production or malfunctioning of IFNs favors the existence of the virus in the host. Among various isoforms of INF, INFα has shown antiviral effects against the SARS-CoV virus, whereas INFβ demonstrated an antiviral effect for both SARS and MERS viruses [74]. The functional relationship between the thymus and different cytokines, including INFs, has shown that thymic cells can initiate the production of cytokines [75]. TECs are considered the source of cytokines production, as the different cytokines and growth factors produced by TECs can modulate the thymic function. Cytokines produced by TECs can be differentiated into four types: proinflammatory cytokines, suppressor cytokines, hemopoietins, and IL-6/IL-7. In line with this, IFNγ can activate the TECs and further enhance the expression of the MHC classes and enhance the production of the IL-6 by the TECs [75]. The high pathogenic nature of SARS-CoV and other coronaviruses makes them sensitive to IFNα/β. There is compelling evidence that the N protein of SARS viruses has been demonstrated as an antagonist for immune and host proteins. Previously, the efficacy and the safety of IFNα in combination with ribavirin (antiviral drug) has been investigated against the SARS-CoV virus in China [76]. Increased secretion of IL-6 in SARS-CoV and MERS-CoV patients has been reported, which shows the pathogenesis in inflammation and viruses [77].

Cytokines have a central role in boosting immune defense against viruses. They initiate, mediate, and regulate the acquired immunity [78]. Studies have shown that INFγ cytokines inhibited the replication of many viruses such as SARS-CoV by blocking viral receptors [79]. INFs bind with viral receptors and block the entrance of viruses into the host cell through the process of endocytosis. In contrast, IL-4 can modulate the activity of INFγ by decreasing the host cell susceptibility and downregulation in viral receptors [80]. In an experiment, Vero E6 cells were exposed to various concentrations of IL-4, IL-10, INFγ, and TNFα after angiotensin-converting enzyme-2 (ACE2) receptor-dependent infection with SARS-CoV. Both IL-10 and TNFα showed no antiviral activity, whereas INFγ or a combination of INFγ and TNFα showed antiviral activity [80]. IL-4 cytokines also showed antiviral activity against SARS-CoV. In another study, HCoV-NL63 virus with ACE2 receptor replication was inhibited by treatment with IL-4 cytokine [81]. These findings illustrate that a combination of IL-4 and IFNγ can stop SARS-CoV replication by decreasing the host cell susceptibility, leading to the downregulation of ACE2 [80]. Apart from this, the malfunctioning of cytokines can suppress or slow down the immune responses by altering the activation of macrophages and DCs, ultimately failing the adaptive immunity [82,83]. SARS-CoV patients have shown overexpression of chemokines and proinflammatory cytokines such as IL-6, leading to pulmonary infections. Additionally, interferon-induced protein-10 (IP-10) and the monocyte chemoattractant protein-1 (MCP-1) chemokines were high in SARS-CoV patients and lung infections [84].

The concentration of IL-6 was high even in the presence of a suppressor of cytokine signaling 3, which generally negatively regulates IL-6 [85]. Additionally, the upregulation of the plasma TNFα cytokine was detected in SARS-CoV patients [86]. Furthermore, it was found that the immune cells producing IL-6 and IL-8 cytokines can weaken the DCs to attack the pathogen, leading to a failed trigger of the adaptive immunity in the lungs of SARS-CoV and MERS-CoV patients [82,83]. SARS-CoV-2 patients have also been found with overexpression of cytokines such as IL-2, IL-7, IL-10, G-CSF, IP-10, MCP-1, macrophage inflammatory proteins-1A, and TNFα. IL-6 receptor antagonists (e.g., tocilizumab and sarilumab) are under phase II/III clinical trial to assess their activity in hospitalized SARS-CoV2 patients [77].

## 6. Role of the Thymus in Humoral Immunity against Coronavirus

B cells are essential elements in the establishment of protective humoral immunity to pathogens. The thymus contains a significant subset of resident CD20+ B cells [87]. The average human and mouse thymus also contains class-switched membrane-IgG+ cells [88,89,90]. Using tissue specimens collected from subjects ranging in age from 5 days to 71 years, it was found that starting during the first year of life, CD138+ plasma cells begin accumulating in the thymic perivascular space where they constitutively produce IgG without the need for additional stimulation [91]. Most thymic IgG-secreting cells produced IgG3 and, to a lesser extent, IgG1. These two IgG subclasses are the most abundant type of IgG produced in viral infections and are most efficient at complement fixation and antibody-dependent cellular cytotoxicity [92,93]. In addition to fully differentiated plasma cells, it was also demonstrated that the thymus contained plasma blasts identified as CD19+ cells expressing IRF4, a key transcription factor driving the plasma cell fate [94]. Thymectomy, however, had a severe adverse effect on residual B lymphocytes: CD19+ B cells [91].

**Figure 7:**
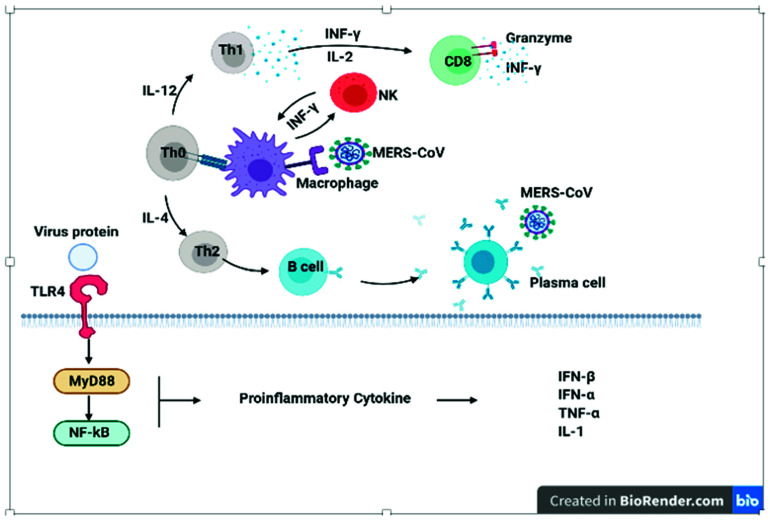
Immune response pathway against MERS-CoV infection. Macrophages present virus antigens to a naive T cell. This process is followed by the activation of T cells. Differentiation induces the production of cytokines specific to the different T cell subsets (Th1, Th2), resulting in the massive production of cytokines. Due to natural killer (NK) cells and cytotoxic T cell (CD8 T cell) activation, these cells produce effective mediators, such as IFNγ and granzyme, to clear a viral infection.

Studies have shown that the absence of the thymus delays the immune response against pathogens due to the depletion of lymphocytes from lymph nodes. Regarding the different antiviral activities driven by the thymus, thymus-dependent antigen-reactive cells are readily needed to produce antibodies to initiate humoral immune responses [95]. The humoral immune responses triggered by the thymic B lymphocytes (Figure 7) can naturalize SARS-CoV-2 [95]. The antibody’s naturalization process is highly active when the antigen is present in the respiratory tract [96], where SARS-CoV-2 and other coronaviruses typically leading to reside. Upon viral infection, the MHC protein of the APCs bind to the specific peptide of the antigen’s epitope followed by interaction with the TCRs to activate B lymphocytes mediated adaptive immune system. TCRs also activate the Th lymphocytes, further boosting the differentiation of B lymphocytes into plasma cells, which have the potential of secreting antibodies against different antigens. Thus, a specific antibody will be produced against the particular antigen presented by APCs. Antibodies neutralize viruses using antibody-dependent cytotoxic cells or NK cells, which can direct the complement proteins to the infected cell for cell lysis. Among five types of antibodies, IgG, IgM, IgA, IgD, and IgE, the first three types are usually associated with antiviral activities [97]. It is known that lymphocyte development occurs in specific lymphoid organs, such as bone marrow and the thymus [98]. Generally, bone marrow is regarded for the production of B lymphocytes, while the thymus is specialized for the development of T lymphocytes. However, B lymphocytes have been seen in the thymus [99], different from peripheral B lymphocytes of bone marrow. Thymic B lymphocytes have the potential to detect several antigens in an extremely high frequency by their B cell receptors, providing higher protection against antigen infections than other B lymphocytes [100]. Regarding the pathogenesis of coronavirus diseases in humans, it has been found that immune responses are triggered by innate immunity [101], followed by the second-line defense, inhibiting viral replications, and most importantly, triggering the adaptive immune responses [102]. Humoral immunity, antibody production, and naturalization have a crucial role in protecting the body against viruses and preventing future recurrence of viral diseases. Although it has been observed that SARS-CoV-2 patients have decreased the number of B, T, Th, and NK lymphocytes [103]. However, it was noticed that the increased size of the thymus is associated with elevated T lymphocyte formation in COVID-infected individuals which is a useful adaptability to COVID-induced lymphopenia. The loss of this thymic role in elderly SARS-CoV-2 patients may lead to a worse prediction [104]. Moreover, SARS-CoV-2 patients have high levels of IL-6 that are responsible for cytokine release syndrome; consequently, cytokine storms might cause death in chronic cases. Acute pathology in severe cases of SARS-CoV-2 is a result of a strong cytokine storm; late IFN response causes the virus to be replicated to avoid the host antiviral reaction and weakens the adaptive immune response [105].

On the other hand, SARS-CoV epitopes of the T and B lymphocytes were thoroughly mapped to analyze the humoral immune responses and the production of different antibodies against a particular virus strain [4]. It has been observed that the total time needed for a specific antibody to develop and can be detected in the serum was 4 to 14 days [74]. Additionally, the antibody produced in a long-lasting stage, about two years, is the IgG-neutralizing antibody. For the other corona family viruses, such as MERS-CoV, the seroconversion detection was after 15 days with the production of IgG antibodies. In SARS-CoV-2 patient serum, the IgM-neutralizing antibody was detected in the first week, whereas the IgG-neutralizing antibody was seen in the second week of infection. Furthermore, sera were collected from five different patients infected with SARS-CoV-2, showing cross-reactivity between SARS-CoV-2 and SARS-CoV. The presence of antibodies confirms the involvement of humoral immunity, especially plasma B lymphocyte, against coronavirus infection. Additionally, in vitro plaque assay from the sera of the patients showed a successful antibody mounting, further confirming the humoral immune responses [106]. B lymphocytes exist in naive B lymphocytes, memory B lymphocytes, and plasma cells that secrete antibodies against viral infections. Plasma cells can produce different antibodies against pathogens, including coronaviruses and complicated epitopes. Plasma cells target the coronavirus epitopes through increased production of different neutralizing antibodies against the specific antigen. When the designed antibodies are injected into a human, they can activate a humoral immune response against coronavirus. Monoclonal antibodies (mAbs) are assortments of different antibodies employed to target different antigens of the spike glycoprotein on the surface of the coronavirus. MERS-CoV antigens are capable of mAbs production based on their ability to trigger humoral immunity [106]. MERS-CoV can adhere to the host cell using the spike glycoprotein on its surface. An enzyme receptor on the host cell called dipeptidyl peptidase-4 (DPP4) is the virus’s entry point. The designed mAbs, m336, are aimed to target the DPP4 and naturalize it by blocking the binding of MERS-CoV to the host cells. Therefore, the design of mAbs against different coronavirus epitopes and stimulating the humoral immunity are urgently required to cope with the viral in the future by sighting deep into the genomics and pattern of infection of SARS-CoV, which shares similar characteristics with SARS-CoV-2 [107]. Recently, ferrets were infected with SARS-CoV and then immunized with monoclonal antibodies with unimmunized control ferrets. Results have confirmed that immunized ferrets were noted with increased, faster, and stronger antibodies than the control animals. Furthermore, IgG production was detected in blood samples of the immunized models, indicating the significance of the IgG antibody in the humoral immune responses [106].

## 7. Conclusions

The thymus is a primary lymphoid organ responsible for generating mature T cells into CD4+ or CD8+ single-positive (SP) T cells, contributing to cellular immunity. Regarding humoral immunity, the thymic plasma cells secrete IgG1 and IgG3 almost exclusively, the two main complement-fixing effector IgG subclasses. Hassall’s corpuscles perform an admirable function. Understanding the endocrine and immune structure and part of the thymus in thymocyte differentiation gives us deep insight into immature T cells, TECs, macrophages, DCs, and thymic B lymphocytes producing humoral immunity and specific cytokine-governing T cell maturation within the thymus. SARS-CoV negatively affects thymocyte development and migration to other lymphoid tissues, followed by cytokine storm-induced chronic inflammation. Understanding the structure and function of SARS-CoV2 helps to develop insight into viral infection with host cells. Thymic hormones have shown promising effects against inflammatory diseases, including SARS-CoV, by promoting the phagocytosis of macrophages and the secretion of antibodies. Recently, various approaches have been adopted to efficiently deliver thymic hormones, such as nanoparticles containing thymus peptides and gene therapy. The immunoregulatory and endocrine function of the thymus activates the immune system by inducing several T lymphocytes and can be effective in preventing inflammatory and virus-related diseases.

## Data Availability

Not applicable.
